# Factorial validity and measurement invariance of the Psychosocial Uncertainty Scale

**DOI:** 10.1186/s41155-021-00190-z

**Published:** 2021-07-30

**Authors:** Mariana Lucas Casanova, Lara S. Pacheco, Patrício Costa, Rebecca Lawthom, Joaquim Luís Coimbra

**Affiliations:** 1 Centre for Career Development and Lifelong Learning, Faculty of Psychology and Education Sciences, University of Porto, Porto, Portugal; 2 Life and Health Sciences Research Institute (ICVS), School of Medicine, University of Minho, Braga, Portugal; 3 ICVS / 3B’s – PT Government Associate Laboratory, Braga, Guimarães, Portugal; 4 Faculty of Psychology and Education Sciences, University of Porto, Porto, Portugal; 5 Faculty of Social Sciences, School of Education, The University of Sheffield, Sheffield, UK

**Keywords:** Psychosocial uncertainty, Coping, Measurement scales, Invariance, Validity

## Abstract

This study presents the development of the Psychosocial Uncertainty Scale (PS-US), which articulates the perception of uncertainty in the social context and its psychological experience. It was validated with a sample of 1596 students and active professionals (employed and unemployed). By randomly dividing this sample in three sub-samples, the following analyses were performed: exploratory factor analysis (sample one: *N* = 827); preliminary confirmatory factor analysis identifying the final version of the scale (sample two: *N* = 382); confirmatory factor analysis (sample three: *N* = 387). Multi-group analysis was used to assess measurement invariance, gender, sociocultural level, and group of origin invariance, by using samples two and three. Group differences were explored with the complete sample through Multiple Indicators and Multiple Causes (MIMIC) Models. Associations between this scale and the Uncertainty response Scale were explored through Structural Equation Modelling. Exploratory and confirmatory analyses’ results showed good internal consistency and overall good psychometric qualities. The scale reached full metric invariance across groups, gender, SCL level and group of origin. Results highlight the sensitivity of the scale towards social vulnerability, proving the existence of sociocultural levels’ effects on experiences of psychosocial uncertainty within working contexts, relationships and community living and self-defeating beliefs; and gender and students versus professionals’ effects on psychosocial uncertainty. Furthermore, the scale associated significantly with Uncertainty Response Scale’s dimensions, specifically with emotional uncertainty, which can be considered a self-defeating strategy. Results suggest that emotional coping strategies, are explained by psychosocial uncertainty by 57%, and so, may have social origins.

**Supplementary Information:**

The online version contains supplementary material available at 10.1186/s41155-021-00190-z.

Most people would agree that contemporary life is rather uncertain. After World War II, the Western world’s industrial and economic development along with concerns for quality and dignity of life allowed the expansion of the welfare state and, with it, a sense and expectation of security. Since the 1980s, sociocultural, historic, economic and political changes seem to have played an important part in generating new forms of subjective uncertainty, thus creating new psychological demands in the process of coping with personal, social and professional contexts that are becoming increasingly unstable and competitive (Bauman, 2001; Coimbra & Menezes, 2009; Sennett, 1998; Tomasik & Silbereisen, 2009). The way each person experiences uncertainty, perceives it in its context and suffers its consequences could be termed as the psychosocial dimension of uncertainty. The current study contributes to research with the development of a new psychological measurement scale that allows the assessment of psychosocial meanings of uncertainty and, so, contributes to theoretical understanding of contemporary uncertainty and the psychosocial challenges it entails.

The psychological mechanism that explains how people deal with the psychological experience of uncertainty can be traced back to attachment relationships. It is the degree of security (or basic trust) (Bowlby, 1980; Erikson, 1963; Marris, 1991, 1996) established in these relationships that enables understanding and coping with uncertainty in a specific manner. By considering the activation of the attachment system in moments of uncertainty, and through a psychosocial view of uncertainty, Marris (1996) defined uncertainty as being created by human needs of order and predictability. These needs are developed through childhood within primordial attachment relationships, through which people construct internal working models that allow understanding and giving meaning to the world, self and others (Bowlby, 1980). Therefore, strategies of prediction and control are used to manage relationships, as well as uncertainty, acting and exerting control on what surrounds us. In this sense, Marris considered uncertainty to be contingent on what interests us to control/predict, on what we are able to predict, and on the confidence in being able to change events (Marris, 1996). Furthermore, Marris draws attention to the way people cope with the environment, by using the same strategies that they use to cope with significant others: “…when grown men and women we are under stress, we revert to treating even the physical world like a parent…” (Marris, 1991, p. 79)—and so, how early attachment experiences influence how people deal with uncertainty in the social context and their takes on order and power.

Based on this conceptualisation of uncertainty and on qualitative research conducted with underprivileged populations as homeless or unemployed people, Marris (1996) concluded the following. Those that live under greater social vulnerability, experience countless consequences of social uncertainty in societies, due to living circumstances and access to resources, from which others are shielded, or have easier access to. The author proposed that there is an unequal distribution of uncertainty and that those powerless to deal with it, are led to adopt self-defeating strategies to manage and control uncertainty, which reinforce their condition and personal sense of inadequacy (e.g*.* the unemployed individual that, after a succession of rejections, lacks the self-confidence necessary to persuade a recruiter of their skills and potential; the homeless person that no longer believes in the possibility of being valuable to society and so avoids seeking job or learning opportunities, believing they will not be capable of dealing with them and so, confirming their own fears).

Various economic, philosophical and sociological studies reinforce a conceptualisation of Western contemporary societies as dominated by fragmentation of communities, frail relationship bonds, as well as labour ones, unstable labour markets and professional atmospheres of distrust and competitiveness (Bauman, 2001; Coimbra & Menezes, 2009). These contribute to individualisation in socialisation, individualism as a style of living and victim blaming as a political validation of inequality, which all contribute to a sense of unpredictability and greater uncertainty for all (Bauman, 2001; Beck, 1992; Marris, 1996; Ryan, 1971/1976; Sennett, 1998).

## Empirical results on uncertainty and associated concepts

Considering the abovementioned social context, empirical research studies have focused on uncertainty or associated concepts. These include materialism as a coping strategy towards feelings of uncertainty (Chang & Arkin, 2002), which could be related with results that prove that uncertainty about the future impacts self-control and leads individuals to “want” choices instead of “should” ones (Milkman, 2012); the perception of risk (Douglas, 1992; Lupton & Tulloch, 2002); the relationship of risk, unemployment and labour legislation (Quilgars & Abbbott, 2000); concepts that connect uncertainty and insecurity with employment, such as “job insecurity” and “employment uncertainty” (de Witte et al., 2016; Mantler et al., 2005; Mohr, 2000) and its family-related outcomes (Mauno et al., 2017); relating uncertainty to the roots of political extremism (Hogg et al., 2013); exploring role ambiguity and role conflict as uncertainty in the workplace (Schmidt et al., 2012); exploring increased labour market uncertainties associated with social and economic change (Obschonka & Silbereisen, 2015); or exploring the impact of economic stressors (also considered as uncertain) on psychological health in Portugal, during the financial crisis (Jesus et al., 2016). Some of these studies use qualitative methodologies and the quantitative ones focus on constructs related to uncertainty (e.g. job insecurity) but not on broader social forms of uncertainty. Other studies sought to explore uncertainty and relate it to social concepts by using the constructs of intolerance of uncertainty (IU) and intolerance of ambiguity (IA): exploring the effects of IU on ethnocentrism (Cargile & Bolkan, 2013), exploring the relationship between right wing authoritarianism and the processing of ambiguous visual stimuli (Duncan & Peterson, 2014).

Within an evolutionary psychology perspective, research has showed environmental uncertainty (analysed through socioeconomic status—SES) led to different responses depending on childhood environment (Griskevicius et al., 2011, 2013; Mittal et al., 2015; Mittal & Griskevicius, 2014). The authors sought to explore how economic uncertainty changes people’s behaviour by altering their sense of control, finding evidence that individuals that grew up in a harsh and unpredictable environment (low SES) tend to perceive environmental threats (here assessed through economic uncertainty) as extrinsic (and so, uncontrollable), while individuals from higher SES will consider it intrinsic (controllable). By assuming they cannot shield from uncertainty, feelings of uncontrollability lead individuals to adopt “fast strategies” (which are defined as evolutionary strategies based on reproductive efforts) (Mittal & Griskevicius, 2014). These contrast with slow strategies, which are employed with extrinsic threats, somatic efforts focused on the “growth and maintenance of one’s body and mind”, including knowledge and skills (Mittal & Griskevicius, 2014, p. 622). However, “fast strategies” are not effective to cope with social threats. Thus, these results showed that people from lower childhood SES presented lower levels of control when faced with an uncertain environment than people from higher childhood SES; that people from wealthier backgrounds felt significantly more control under uncertainty than in the control condition, while people from poorer backgrounds reported less control; and that economic uncertainty influenced the personal sense of control but not the perception of others’ sense of control, and so all participants perceived others as having less control under uncertainty, regardless of their own SES. Furthermore, results showed that perceptions of control mediated the effect of uncertainty on impulsive behaviour and on persistence, depending on childhood SES. So, people from poorer childhoods (more exposed to uncertainty) became more impulsive and less persistent because they felt less control. These results seem quite relevant, although we do not adopt an interpretation of them based on an evolutionary perspective but on an ecological and developmental one that considers historical and social dimensions of psychological experiences (Bronfenbrenner, 2005; Gergen, 1996). These childhood experiences must be framed within their social and historical context, along with the quality of children’s psychological experiences, relationships and meaningful contexts—the family, neighbourhood, school and community. So, by considering these strategies as potentially self-defeating ones, they may be explained by individual’s life-long experiences, while also acknowledging their capacity for agency and to intervene in their immediate contexts. Therefore, the experience of uncontrollability experienced by individuals from vulnerable social groups becomes of the utmost importance to understand their relationship with the future, possible disbelief in it and, therefore, a tendency to adopt self-defeating strategies or to act impulsively due to the actual powerlessness of some to control the present and the future (Marris, 1996; Prilleltensky, 1994).

Tomasik and Silbereisen (2009) created a scale that focuses on demands of social change (on work and family life) due to globalisation and individualisation and this scale has allowed to explore group differences related to resources such as employability status, political contexts (Tomasik & Silbereisen, 2009), career planning (Lechner et al., 2016), religiosity (Lechner et al., 2013) and the impact of the global financial crisis (Tomasik & Silbereisen, 2016).

### The psychosocial uncertainty scale: the present study

As previously described, even though there are quantitative studies on the experience of uncertainty, these either focus on a trait approach, of levels of tolerance to uncertainty, or combine uncertainty with other constructs, analysing a specific aspect or context of uncertainty (e.g., employment uncertainty; economic uncertainty). On the other hand, Tomasik and Silbereisen (2009) reflects uncertainty by expressing perceived demands of the social context. Therefore, we decided to create a measure that could directly focus on the psychosocial experience of uncertainty as reflecting individual experiences of broad social forms of uncertainty and so further research in this scope.

Inspired by Marris’ proposal and taking into account the previous studies and theoretical contributions, a set of 22 items was created for the Psychosocial Uncertainty Scale. The scale intends to reflect how uncertainty is perceived and experienced in contemporary Western societies, combining its psychological meaning with its social, cultural and political origins. Thus, each item articulates the perception of uncertainty in the social context (e.g. the labour market, or community living) and how individuals experience it psychologically: with concern, affecting decision-making, with distrust towards others or through feelings of disempowerment. Thus, the scale’s items reflect Marris’ (1991, 1996) proposal on how self-defeating strategies emerge when coping with uncertainty in a society in which it is unequally distributed.

Factor structure, validity and reliability results will be presented by using three subsamples drawn from a sample of 1596 participants, as well as multi-group measurement invariance and invariance across gender, sociocultural levels and group of origin (students versus professionals) (students from technical training to master versus active professionals, employed or unemployed). Furthermore, the effects of these demographic variables will be explored in order to establish the scale’s potential and validity. We will investigate the hypothesis that individuals from underprivileged social groups (women, lower sociocultural levels) experience greater levels of psychosocial uncertainty in their lives. Finally, considering Marris’ proposal of an unequal distribution of uncertainty and of the power to cope with it (that leads vulnerable individuals to adopt self-defeating strategies), and that these inequalities are socially created, we will explore the relationship of PS-US with the Uncertainty Response Scale—URS (Greco & Roger, 2001) and its dimensions, hypothesising that the PS-US may contribute to variation in the URS. These analyses intend to demonstrate the advantages of an integrated study of uncertainty, considering psychosocial dimensions and constraints in the way people deal with uncertainty.

## Method

### Procedures

Higher Education Institutions, Training Companies and Centres were contacted, inviting their collaboration in the distribution of the study weblink to students and former students. The aim was to achieve a national sample composed by adults, both students and active professionals, through snowball procedures. There were no a priori sample size calculations, though the purpose was to roughly obtain: 50% students and 50% active professionals; a diversity in this group regarding the employment status; and at least 10 participants per item considering the number of items in the scales’ being used (which would entail a minimum of 470 participants).

To avoid biases that could influence responses to both scales, it was decided to randomly mix items from the PS-US and from the URS (Kline et al., 2000). For that reason, the same Likert scale was used in both instruments. Moreover, both scales included inverted items to avoid halo effects. The online questionnaire included a brief explanation of the research and clear, specific and univocal instructions, guaranteeing confidentiality and anonymity.

### Participants

The study sample comprises 1596 participants: 55.6% students and 44.4% professionals (31.5% employed and 12.8% unemployed), 70.7% females, age average of 26.98 (standard deviation 8.658). Concerning sociocultural level distribution (SCL), 36.1% are from middle-lower/lower classes, 19.9% middle class and 44% middle-upper/upper class. This majority of middle and upper-class individuals is explained by the fact that the SCL level was calculated by considering educational levels. Moreover, this form of data gathering influences access to other population segments (e.g. with less digital access). Table 1 presents the composition of the three subsamples extracted from the global sample. There are no significant differences between these samples in what concerns these sociodemographic variables, except for gender distribution in the sample used for CFA2. Within these variables, there are a few missing values (m.v.) for age: EFA (sample 1) six m.v.; CFA1 (sample 2) five m.v.; CFA2 (sample 3) nine m.v.

**Table 1:** Demographic characteristics by sample

	Gender		Sociocultural levels (SCL)			Group of origin		Age
	Male	Female	Lower	Middle	Upper	Students	Active professionals	
Complete sample (*N* = 1596)	468 (29.3%)	1128 (70.7%)	576 (36.1%)	318 (19.9%)	702 (44.0%)	888 (55.6%)	708 (44.4%)	26.88 (8.61)
EFA (Sample 1) (*N* = 827)	255 (30.8%)	572 (69.2%)	310 (37.5%)	162 (19.6%)	355 (42.9%)	475 (57.4%)	352 (42.6%)	27.21 (9.14)
CFA1 (Sample 2) (*N* = 382)	121 (31.7%)	261 (68.3%)	126 (33%)	76 (19.9%)	180 (47.1%)	194 (50.8%)	188 (49.2%)	26.67 (8.2)
CFA2 (Sample 3) (*N* = 387)	92 (23.8%)	295 (76.2%)	140 (36.2%)	80 (20.7%)	167 (43.2%)	219 (56.6%)	168 (43.4%)	26.35 (7.77)
Sample Comparison *X*^2^ (df)	7.68 (2)		2.75 (4)			4.87 (2)		Anova for age: F(2, 1573) = 1.44, *p* = .237, *η*2 =.002 There are no differences between samples in terms of age
Sample Comparison p value	.02		.60			.09		

Gender; sociocultural status and group of origin characterised by *n* and (%); age characterized as M (SD)

### Materials

#### Sociodemographic Questionnaire

Composed of sociodemographic and situational questions pertinent for sample characterisation, namely gender and years of schooling. The sociocultural level was based on years of schooling and professional situation of the active professionals and on years of schooling and professional situation of the parents of students.

#### Development of the Psychosocial Uncertainty Scale

Twenty-two items were generated, by transforming abstract and conceptual aspects in observable statements that may reflect attitudes, thoughts or emotions, carefully worded in a clear, specific and univocal fashion, including one single idea, with appropriate language, to guarantee variability. Given the fact that the underlying construct involves, on the one hand, the perception of uncertainty in the social context, and on the other hand, the way individuals give meaning to it and experience its consequences, item formulation was complicated by the construct’s very nature of interaction (Clark & Watson, 1995). The items formulated combine psychological and social dimensions of uncertainty by identifying psychological consequences within work (e.g. Because of the characteristics of the labour market, I feel increased difficulties in making decisions.); within the relational/communitarian context as a whole (e.g. The competition that exists in nowadays societies makes me feel I cannot trust others.), but also self-defeating beliefs about uncertainty (e.g. In spite of the unpredictability of contemporary life, I feel I can plan my future.). The original items in Portuguese and an English translation can be consulted in Supplementary Material—Table C1.

The items were discussed with a panel of expert researchers in Psychology. A five-points Likert scale was used with the purpose of not over complicating the process of response, considering the intention to gain access to a broad sample in terms of schooling years (Clark & Watson, 1995). Ten interviews were performed with individuals that corresponded to the target population in terms of age, occupational situation, schooling and sociocultural level. In consequence, items were reviewed so to make them as clear and accessible as possible. Through this process, facial and content validity were assessed in terms of item interpretation, adequate formulation regarding the population and variables under study.

#### Uncertainty Response Scale (URS, Greco & Roger, 2001)

Composed of 25 items in its Portuguese version, it contains three factors: emotional uncertainty with 11 items (perceiving uncertainty as a stressor and responding to it with anxiety and sadness – α = .91); cognitive uncertainty with 6 items (planning, clarifying and gathering information as a personal need in order to control uncertainty – α = .82.) and desire for change with 8 items (reflecting a sense of enjoyment and desire in what concerns change and unexpectedness – α = .88) (Lucas Casanova et al., 2019).

### Data analysis

**Fig. 1:**
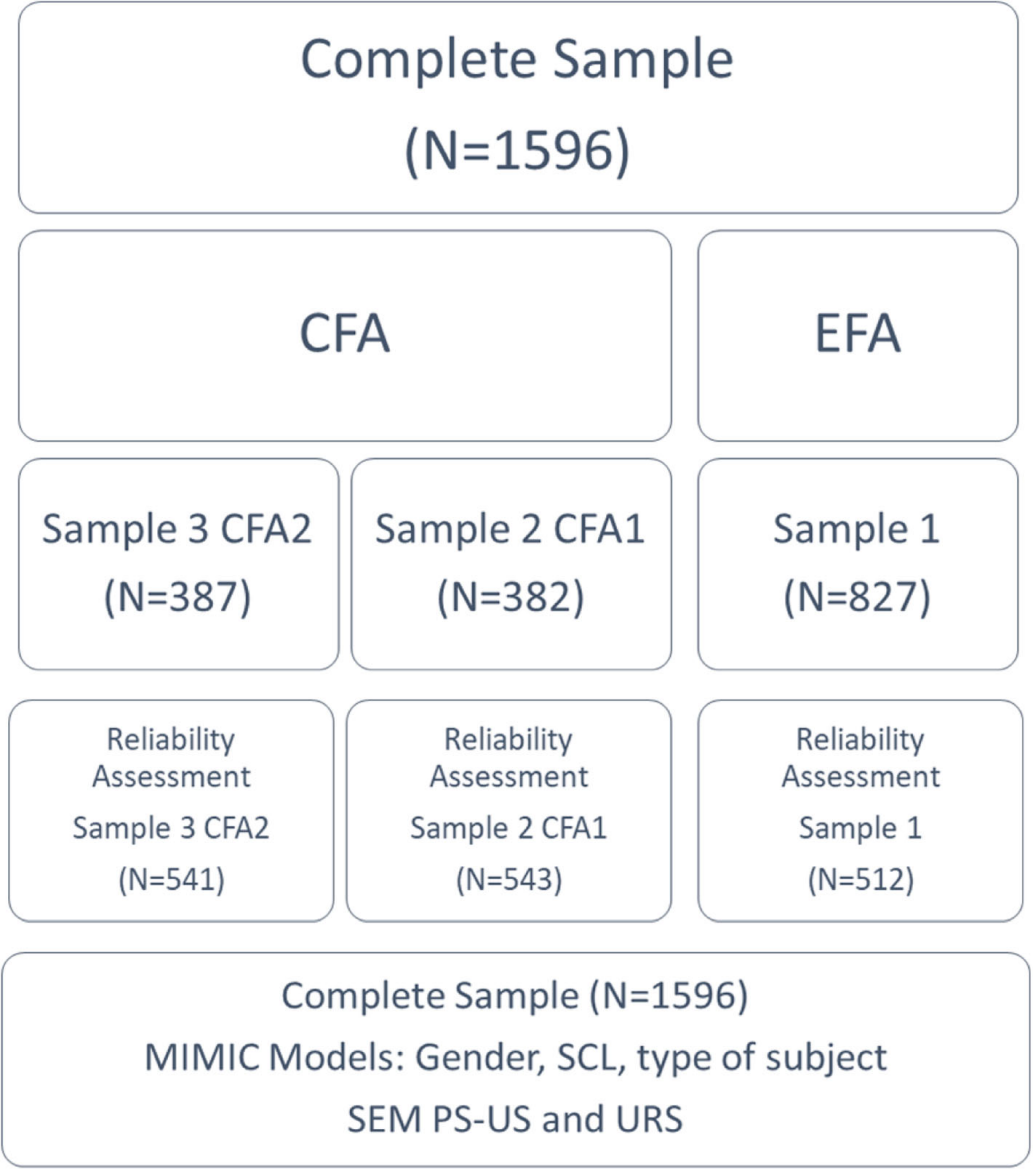
The procedure for data analysis

Half of the original sample was used for the exploratory factor analysis (sample 1). The other half was randomly divided in two samples, so that one was used for a preliminary confirmatory factor analysis (sample 2) of PS-US (considering its exploratory nature) and the confirmatory factor analysis (sample 3), and both were used for multigroup analysis of invariance. These samples were also used for reliability analyses. Figure 1 presents the procedure for data analysis. Descriptive statistics, exploratory factor analysis and multivariate analyses of variance were completed with IBM SPSS Statistics 24; confirmatory factor analyses, multi-group analyses, MIMIC Models and Structural Equation Modelling of the relationship between the two scales were performed with IBM SPSS Amos 24. The whole sample was used for the MIMIC Models and to explore the relationship of the PS-US with the URS. Three missing values (m.v.) were identified in the whole sample in two items of the PS-US (1 m.v. in item 1 and 2 m.v. in item 2) but all participants were kept. Thus, when using IBM SPSS Statistics 24, analyses excluded missing values cases’ listwise and when using IBM SPSS Amos 24, m.v. were imputed using its data imputation features, considering the CFA’s structure.

## Results

Supplementary information (appendix A) presents PS-US’ items descriptive statistics (range, means, medians, standard deviations, skewness and kurtosis) to assess the accomplishment of assumptions for the analyses performed. Data presents adequate scores of skewness and kurtosis (Kline, 2005)—highest skewness and kurtosis found in item 19 (sk = − 1.19 and ku = 1.61). The analyses identified some univariate outliers, but it was decided to keep them. Normality, linearity, homogeneity of variance-covariance matrices and multicollinearity analyses were performed to test relevant assumptions and no serious violations were identified. Examinations were also performed on univariate and multivariate outliers and while some were detected, none were deleted.

### Exploratory factor analysis

Given the exploratory nature of this scale, the 22 initial items were previously analysed using different strategies of extraction and rotation in early stages of this research (Casanova, 2010; Casanova et al., 2010). However, considering the moderate correlations found between factors and to follow a reflective factorial model of analysis, as confirmatory factor analysis (CFA) (using only covariance between variables and therefore reducing the variability of errors), the authors chose to further explore results through principal axis factoring, with an oblique rotation—Direct Oblimin (Tabachnick & Fidell, 1996/2007).

Most intercorrelations between items proved moderate and low with just a couple of items over .5 (Clark & Watson, 1995)—items 7 and 8 present the highest correlation .58). As expected, the anti-image diagonal revealed values above 0.5, except for item 2, which is later eliminated. Bartlett’s sphericity test was significant but the test’s sensitivity to sample size must be considered (Tabachnick & Fidell, 1996/2007). Kaiser-Meyer-Olkin proved satisfactory (0.86). Following the scree test analysis and the values of initial eigenvalues (higher than one), we tested a solution of three factors using a conventional exclusion criterion of 0.30, achieving a solution of 13 items distributed as presented in supplementary information—Appendix B – Table B1. In this appendix, Table B2 presents eigenvalues and variance explained and correlations between factors and Table B3 presents each factor’s mean, standard deviation and correlations between factors. However, items 7 and 8 saturated in two factors. Nevertheless, considering the exploratory nature of the scale and the complexity of the items’ underlying construct, it was decided to keep them to analyse their behaviour in these analyses. Indeed, as Marsh et al. (2009), p. 447) have stressed, even though “…there are advantages in having ‘pure’ items that load on a single factor, this is clearly not a requirement of a well-defined, useful factor structure”. So, considering statistical results, theoretical assumptions that led to the creation of these items, as well as its content (and facial validity analysis from experts), we decided to keep them in the factor related to work experiences.

The three factors previously briefly presented were interpreted considering the content of the items and the construct at their origin. Factor one was named Psychosocial Consequences at Work given this dimension expresses daily concerns with work, which are perceived as consequences of uncertainty. This dimension may reflect an unequal distribution of uncertainty in terms of employment and working environments (Bauman, 2001; Marris, 1996; Sennett, 1998), revealing how work constrains the access to living circumstances or resources that may facilitate dealing with uncertainty. Factor two was labelled Psychosocial Consequences within Relationships and Communities since its items describe experiences of uncertainty within relationships (or inscribed in broader social structures such as communitarian living), which seem to be perceived and experienced as uncontrollable and negative. These experiences may be related to an intensification of distrust towards the abstract social “other” (Beck, 1992), associated with a community deficit in contemporary social relationships (Coimbra & Menezes, 2009). This factor may reflect forms of individualism and of individualisation as a form of socialisation, which ultimately generates insecurity, distrust and competitiveness in relationships, which all create uncertainty, contributing to the phenomenon of victim blaming (Ryan, 1971/1976). Factor three was titled Self-defeating Beliefs^1^, transmitting a personal belief of not being able to manage the future and uncertainty, allowing to identify individuals that do not believe in their capacity to manage uncertainty and control the future, which can be considered as a self-defeating belief and eventually may lead to the adoption of self-defeating strategies.

### Preliminary confirmatory factor analysis

Considering that this is a new scale and so, an exploratory study, a preliminary confirmatory factor analysis (CFA) was performed to achieve a satisfactory structure. Using the maximum likelihood estimator, a CFA was performed. The quality of factorial adjustment was evaluated by the main indices and reference values proposed in the literature (Brown, 2006): chi-square test, chi-square/degrees of freedom between 1 and 2, Comparative Fit Index—CFI above .90 and root mean square error of approximation—RMSEA, *P*[rmsea ≤ 0.05] below .80. The three-factor model of PS-US achieved low fit as showed in Table 2 (Model A): χ^2^/df = 4.2, CFI = .85, TLI = .82; RMSEA = .09; *P*[rmsea < .001]; SRMR = .075. Consequently, all items with standardised regression weights above .55 were conserved, thus achieving a significance of .31 (almost one third of item variance).

**Table 2:** Goodness of fit indices for the model of the confirmatory factor analyses for the PS-US

CFA1 (*N* = 382)											CFA2 (*N* = 387)									
	χ^2^ (df)	p value	χ2/ df	CFI	TLI	RMSEA	LO 90	HI 90	P (RMSEA)<.05	SRMR	χ^2^ (df)	p value	χ2/ df	CFI	TLI	RMSEA	LO 90	HI 90	P (RMSEA)<.05	SRMR
Model A	260 (62)	*p* < .001	4.2	.85	.82	.092	.080	.10	< .001	.075										
Model B1	113 (32)	*p* < .001	3.6	.93	.90	.082	.066	.098	.001	.057										
Model B2	67 (31)	*p* < .001	2.2	.97	.95	.055	.037	.073	.293	.047	90 (31)	*p* < .001	2.9	.94	.92	.070	.054	.087	.024	.042

Subsequently, the model achieved a good fit (model B1): χ^2^/df = 3.56, CFI = .93, TLI = .90; RMSEA = .08; *P*[rmsea < .05]; SRMR = .057. Nevertheless, modification indices (with a threshold of 11) proposed correlating the errors of items 3 and 10 (both from Psychosocial Consequences at Work). So, it was decided to include this error correlation in the final model since these items share theoretical content, leading to the following results (model B2): χ^2^/df = 2.17, CFI = .97, TLI = .95; RMSEA = .06; *P*[rmsea < .05]; SRMR = .047. These two models were compared, proving model’s B2 better fit: (χ^2^ (1) = 46.6, *p* < .01), along with a lower Model Expected Cross Validation Index—MECVI (.42 vs. .31). Thus, we reached the final solution for the PS-US and confirmed its internal structure validity with sample two. Table C1 in Appendix C of supplementary information presents the distribution of items per factor and their standardised regression weights for this final version.

### Confirmatory factor analysis

Considering this scale’s exploratory nature, another CFA was performed with sample three to confirm its internal structural validity, and the model achieved a good fit: χ^2^/df = 2.90, CFI = .94, TLI = .92; RMSEA = .07; *P*[rmsea < .01]; SRMR = .042. To facilitate presentation of results, Table 2 compares results from both CFA’s performed.

### Multi-group invariance

After confirming the scale’s internal structural validity and final solution, a multi-group invariance analysis was performed by comparing samples two and three. Results corroborate the factor structure of the scale through its good fit in both samples, proving the configural invariance of the model: χ^2^/df = 2.5, CFI = .96, TLI = .93; RMSEA = .05; *P*[rmsea > 0.05].

Subsequently, the unconstrained model was compared with models in which measurement weights, intercepts, structural covariances and measurement residuals are gradually constricted. By using the Chi-squared test comparison, statistical significance was found to prove metric invariance. Subsequent invariance levels were not proven through Δ χ2 test (and respective *p* value). Nevertheless, chi-square is also reliant on sample size and so other indices are also presented and account for loading invariance: Δ CFI, < or equal to − .01; Δ RMSEA (< .015), and Δ standardised root mean square residual (SRMR) < .025 (Chen, 2007; Cheung & Rensvold, 2002). Except for scalar invariance (*p* = .045), all Δ χ^2^ tests are non-significant and all results for Δ CFI, Δ RMSEA and Δ SRMR provide support for strong invariance. Thus, if scalar invariance is assumed (considering for Δ CFI, Δ RMSEA and Δ SRMR), results support structural invariance. Further invariance studies may prove useful for the assertion of the scale’s invariance. Table 3 presents results for multi-group invariance between these two samples.

**Table 3:** Goodness-of-fit measurement invariance tests for PS-US

Invariance level	Definition	Model	χ2	df	Δ χ2	Δ df	p	CFI	RMSEA	Δ CFI	Δ RMSEA	Δ SRMR
Multi-group invariance tests comparing samples used for CFA1 and CFA2												
Configural invariance	Same factor structure	M1	157	62				.96	.045			
Metric invariance	Same factor structure and factor loadings	M2-M1	159	69	2.39	7	.94	.96	.041	.002	< .015	< .005
Scalar invariance	Same factor structure, factor loadings and intercepts	M3-M2	178	79	18.7	10	.05	.95	.040	− .004	< .015	< .005
Error variance invariance	Same factor structure, factor loadings and error variances	M4-M3	185	85	7.50	6	.28	.95	.039	− .001	< .015	< .005
Structural invariance	Same factor structure, factor loadings, error variances and factors’ covariance	M5-M4	192	95	6.80	10	.74	.95	.037	.002	< .015	< .005
Multi-group invariance tests for Gender (using samples CFA1 and CFA2)												
Configural invariance	Same factor structure	M1	131	62				.97	.038			
Metric invariance	Same factor structure and factor loadings	M2-M1	135	69	4.66	7	.70	.97	.036	.001	< .015	< .005
Scalar invariance	Same factor structure, factor loadings and intercepts	M3-M2	184	79	49	10	< .001	.95	.042	− .019	< .015	< .005
Error variance invariance	Same factor structure, factor loadings and error variances	M4-M3	193	85	9.07	6	.17	.95	.041	− .001	< .015	< .005
Structural invariance	Same factor structure, factor loadings, error variances and factors’ covariance	M5-M4	214	95	20.8	10	.02	.94	.041	−.006	< .015	< .005
Multi-group invariance tests for Sociocultural Level–SCL (using samples CFA1 and CFA2)												
Configural invariance	Same factor structure	M1	177	93				.96	.034			
Metric invariance	Same factor structure and factor loadings	M2-M1	192	107	15	14	.38	.96	.032	.00	< .015	< .005
Scalar invariance	Same factor structure, factor loadings and intercepts	M3-M2	230	127	38	20	.01	.95	.033	− .01	< .015	< .005
Error variance invariance	Same factor structure, factor loadings and error variances	M4-M3	258	139	28	12	.01	.94	.034	− .01	< .015	< .005
Structural invariance	Same factor structure, factor loadings, error variances and factors’ covariance	M5-M4	287	161	29	22	.15	.94	.032	.00	< .015	< .005
Multi-group Invariance tests for students versus professionals (using samples CFA1 and CFA2)												
Configural invariance	Same factor structure	M1	147	62				.96	.042			
Metric invariance	Same factor structure and factor loadings	M2-M1	149	69	2	7	.97	.96	.039	.002	< .015	< .005
Scalar invariance	Same factor structure, factor loadings and intercepts	M3-M2	199	79	50	10	< .001	.94	.045	− .018	< .015	< .005
Error variance invariance	Same factor structure, factor loadings and error variances	M4-M3	202	85	3	6	.74	.94	.043	.002	< .015	< .005
Structural invariance	Same factor structure, factor loadings, error variances and factors’ covariance	M5-M4	222	96	20	11	.04	.94	.042	−.003	< .015	< .005

### Multi-group invariance analysis—gender

Multi-group analysis on gender was tested for the scale by joining the same two samples previously used. Table 3 also presents results for Gender invariance, providing evidence for configural invariance: χ^2^/df = 2.12, CFI = .97, TLI = .95; RMSEA = .038; *P*[rmsea ≤ .05 = .985]. Full metric invariance was verified through the Δ χ^2^ test *p* value, as well as through Δ CFI, Δ RMSEA and Δ SRMR. However, scalar invariance was not proven through the Δ χ^2^ (*p* < .001) and Δ CFI, despite acceptable values for Δ RMSEA and Δ SRMR, which could be related to the unbalanced character of the sample in terms of gender.

### Multi-group invariance analysis—sociocultural level

Invariance according to three sociocultural levels was tested (low, middle, upper). Δ χ^2^ test is non-significant for metric invariance but not for scalar invariance. Nevertheless, results for Δ CFI, Δ RMSEA and Δ SRMR provide support for subsequent levels of invariance. So, full metric invariance was proven, along with partial scalar invariance, which indicates that it could benefit from further studies. Table 3 presents results for sociocultural level (SCL) invariance.

### Multi-group invariance analysis—students versus professionals

Invariance regarding the group of origin (students versus professionals) was explored to subsequently test for potential effects of this variable on psychosocial uncertainty. Table 3 presents these results, providing support for full metric invariance through the Δ χ^2^ test *p* value, Δ CFI, Δ RMSEA and Δ SRMR. Scalar invariance, however, was not proven (Δ χ^2^ test *p* < .001; Δ CFI > .01) though acceptable values for Δ RMSEA and Δ SRMR were found and so, invariance regarding populational groups should be additionally explored.

### Reliability

Factors’ internal consistency was assessed by alpha coefficient for the three samples and composite reliability (CR) for the two samples used for multi-group analysis, as well as through the average variance extracted (AVE), achieving values considered to be satisfactory in exploratory research (Hair Jr. et al., 1998): Psychosocial consequences at work presented an alpha (α) of .80 for the exploratory factor analysis (EFA) sample (with 6 items), a .82 α, a CR of .80 and an AVE of .45 for the CFA 1 sample (with 5 five items), a .79 α, a CR of .78 and an AVE of .42 for the CFA 2 sample; psychosocial consequences within relationships/communities presented α of .71 for the EFA sample (with 5 items), a .73 α, a CR of .73 and an AVE of .48 for the CFA 1 sample (with 3 items), and a .69 α, a CR of .69 and an AVE of .43 for the CFA 2 sample; self-defeating beliefs presented a .67 α for the EFA sample (with 2 items, which remained the same), a .63 α, a CR of .64 and an AVE of .47 for the CFA 1 sample, and a .61 α, a CR of .61 and an AVE of .44 for the CFA 2 sample.

Construct validity was supported through factorial validity, which reinforces the specification and distribution of items in the scale. Even though the third dimension of PS-US (self-defeating beliefs) may be further developed in order to strengthen its psychometric qualities, consistency in results from both exploratory and confirmatory factor analyses, as well as the factor’s CR and AVE acceptable results support its potential (Cortina, 1993). In addition, despite lower results in coefficient alpha and CR of this factor, inter-item correlation fitted recommended results that deem this alpha acceptable (Briggs & Cheek, 1986). So, convergent validity was assessed through factor loadings (standardised regression weights) and inter-item correlations, achieving acceptable results. Discriminant validity of each factor was assessed by comparing each factor’s AVE to the square of correlations between factors. Given that these were inferior to the AVE of the factors involved, discriminant validity was found between all subscales.

### Effects of sociodemographic variables on psychosocial uncertainty

In order to demonstrate the value of the PS-US and the connection between psychological dimensions of uncertainty and social ones, this study’s final step was to assess the effect of three sociodemographic variables in psychosocial uncertainty: gender, sociocultural level (SCL) and group of origin (students and professionals). For each of these groups, full metric invariance was proved along with partial scalar invariance for SCL. Nonetheless, it was decided to explore the effects of these sociodemographic variables on psychosocial uncertainty, considering the promising results for general multi-group invariance and the need to further explore this scale’s potential. Therefore, effects were tested through Multiple Indicators and Multiple Causes (MIMIC) Models (Brown, 2006; Kline, 2005) in which all sociodemographic variables were added to the model of the scale, allowing for an analysis that considers the full model, error-variance and the adjusted effects of these predictor variables on the latent factors. The complete sample was used for these analyses as well as all the subsequent ones.

**Fig. 2:**
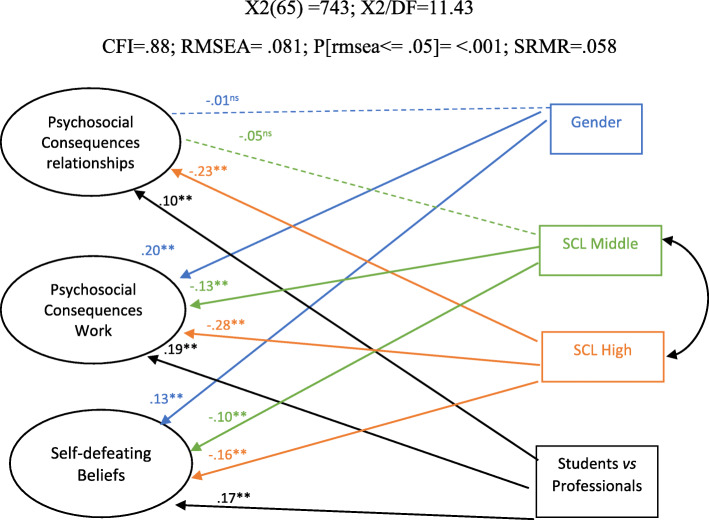
Mimic—the effect of gender, sociocultural level (SCL) and group of origin on the PS-US

The hypothesis that orients these analyses is that socially vulnerable groups (in this case, women and individuals from lower SCL) experience higher levels of psychosocial uncertainty in all its dimensions: in the context of work, social relationships and community life, as well as self-defeating beliefs on the possibility to cope with uncertainty, similarly to what was found in terms of adoption of emotional coping strategies towards uncertainty, usually considered in the literature to be maladaptive (Lucas Casanova et al., 2019), thus exploring the scale’s concurrent validity. We do not expect differences in terms of psychosocial uncertainty between students and professionals. However, different occupational situations may entail different experiences regarding work and so, professionals may experience more uncertainty in the form of job insecurity and precarity (de Witte et al., 2016; Mantler et al., 2005; Mohr, 2000). Figure 2 presents the following results.

Results demonstrate a significant effect of gender on two of the factors: psychosocial consequences at work (β = .204; *p* < .001) and self-defeating beliefs (β = .131; *p* < .001), confirming that women (identified as 1, and men as 0) experience more strongly the psychosocial consequences of uncertainty at work and develop self-defeating beliefs on coping with uncertainty.

Regarding students (identified as 0) versus professionals (identified as 1), the same tendency was found: psychosocial consequences at work (β = .191; *p* < .001) and self-defeating beliefs (β = .17; *p* < .001); and psychosocial uncertainty within relationships/communitarian living (β = .102; *p* < .001). Thus, professionals may experience their environmental circumstances as more uncertain than students, mostly in terms of their relationship to the labour market (in which some students may be already integrated while others not), potentially generating self-defeating beliefs on their capacity to cope with uncertainty.

For SCL, middle and high SCL participants were compared with low SCL participants. Results show that participants from middle and upper SCL experience less psychosocial uncertainty than participants from lower SCL: psychosocial consequences at work for high SCL (β = − .284; *p* < .001); self-defeating beliefs for high SCL (β = − .155; *p* < .01); psychosocial uncertainty within relationships/communitarian living for high SCL (β = − .226; *p* < .001); psychosocial consequences at work for middle SCL (β = − .129; *p* < .001); self-defeating beliefs for middle SCL (β = − .101; *p* < .05); results were non-significant for psychosocial uncertainty within relationships/communitarian living for middle SCL when comparing with low SCL. In a second step, participants from middle SCL were compared with high SCL. Significant differences were found for psychosocial uncertainty within relationships/communitarian living, in which high SCL present lower scores (β = − .169; *p* < .001), as well as for psychosocial consequences at work in which participants from high SCL present lower scores than the ones from middle SCL (β = − .126; *p* < .005).

This means that the low and middle classes suffer psychosocial consequences of uncertainty more intensely than the upper class. These results support the hypothesis that the upper classes benefit from environmental experiences with less uncertainty, experiencing protection from the consequences of uncertainty at work and in relationships/communitarian living, maintaining a higher sense of security.

### Associations between PS-US and URS

To explore PS-US items’ sensitivity and the scale’s convergent and divergent validity, the relationship between the PS-US and the URS was explored. The URS assesses coping with uncertainty and, so both scales share the basic construct of uncertainty, so they were expected to correlate. Nevertheless, the scales have different core constructs and so this analysis is relevant for the assessment of divergent validity.

Correlations between the two scales were performed through a Structural Equation Model (SEM). As expected, its dimensions were significantly correlated, contributing to the analysis of convergent validity. All correlations are significant at a *p* < .001: psychosocial consequences at work correlate significantly with Emotional Uncertainty (.72), Cognitive Uncertainty (.30) and Desire for Change (− 19); Psychosocial consequences within relationships/communities correlate significantly with Emotional Uncertainty (.60), Cognitive Uncertainty (.35) and Desire for Change (− .13); and Self-defeating beliefs correlate significantly with Emotional Uncertainty (.32), Cognitive Uncertainty (− .29) and Desire for Change (− .35).

The moderate/strong positive associations between emotional uncertainty and psychosocial consequences of uncertainty at work and within relationships reinforce two of the theoretical propositions previously presented: that working and communitarian environments are constrained by uncertainty (Bauman, 2001; Beck, 1992); that the constraints exercised by uncertainty in these social contexts, may have a negative impact in individuals’ reactions, leading them to resort to self-defeating strategies, in this case emotional coping strategies (Marris, 1996). Moreover, the dimension self-defeating beliefs presents a negative weak correlation with desire for change, suggesting an opposition between positive attitudes towards uncertainty and adopting self-defeating beliefs/strategies. Therefore, the scales demonstrate to complement each other in the analysis of how individuals cope with uncertainty within the social domain, proving to be useful instruments in this research area, which contributes to the assessment of its criterion and convergent validity.

Considering that the PS-US reflects a social environment of uncertainty, it was hypothesised that coping with uncertainty could be explained by psychosocial uncertainty. Given this is a cross-sectional study, we explored which dimensions of psychosocial uncertainty were better predictors of emotional uncertainty, cognitive uncertainty and desire for change through a SEM. The dimensions of PS-US were identified as independent variables and each of the URS dimensions as dependent variables.

**Fig. 3:**
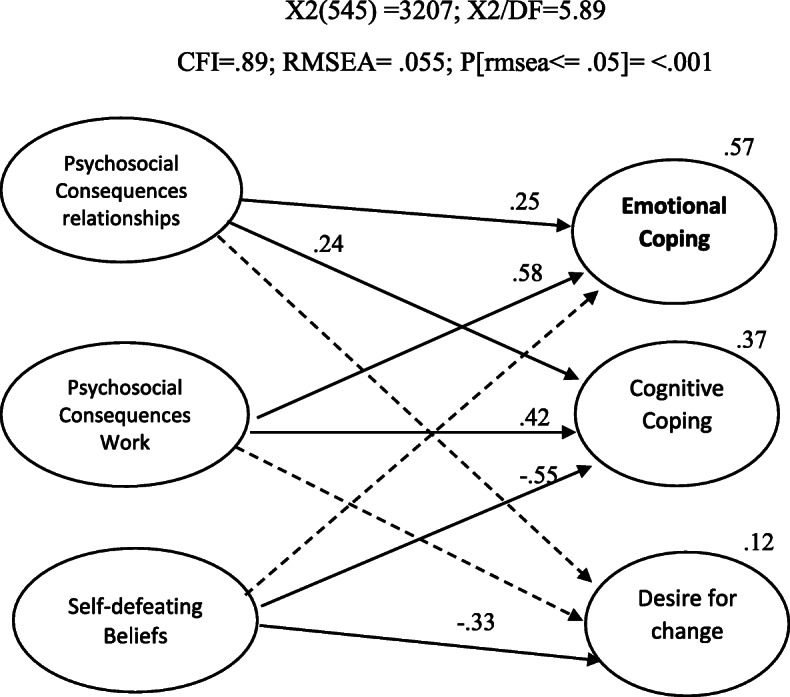
SEM—psychosocial uncertainty and emotional coping strategies towards uncertainty

Given the size of the sample, it can be considered that the model achieved acceptable quality of adjustment considering the following indices: χ^2^/df = 5.89, CFI = .89, TLI = .88; RMSEA = .055; *P*[rmsea ≤ 0.05] < .001. Figure 3 presents these results, which indicate that psychosocial uncertainty explains the variance of emotional uncertainty by 57%, cognitive uncertainty by 37% and desire for change by 12%. Additionally, the significance of the effects of psychosocial uncertainty on coping with uncertainty was tested through bootstrapping (bias-corrected two-tailed confidence intervals). Results demonstrate that psychosocial consequences of uncertainty at work have a significant positive effect on emotional uncertainty (β = .583; *p* < .005) and on cognitive uncertainty (β = .419; *p* < .005). So, the experience of uncertainty at work contributes to adopting emotional and cognitive strategies of coping with uncertainty. Similarly, psychosocial uncertainty within relationships /community living has a significant positive effect on these variables: emotional uncertainty (β = .255; *p* < .005) and on cognitive uncertainty (β = .239; *p* < .005). On the contrary, self-defeating beliefs on coping with uncertainty have a negative significant effect on cognitive uncertainty (β = − .55; *p* < .005) and on desire for change (β = − .334; *p* < .005).

These results seem to endorse the hypothesis that individuals’ environmental circumstances may explain emotional responses (considered maladaptive). Therefore, these environmental circumstances may affect the way people give meaning to uncertainty within the social context, how they experience its consequences in working and communitarian contexts and, ultimately, how they cope with uncertainty. We can interpret this as an indication that the sources of some forms of uncertainty (and how people signify and experience them) can be found in socioeconomic, historical and political contexts, as Marris (1996). Nonetheless, the model would have to be explored through longitudinal studies to confirm any forms of causal interpretations.

Furthermore, psychosocial uncertainty’s consequences within work proved to be the variable that better explains emotional uncertainty. To interpret this result, it is important to consider previous results on emotional uncertainty as being significantly different among social groups (Lucas Casanova et al., 2019), showing that individuals from lower SCL would present higher levels of emotional uncertainty, as well as women. Together, these results lend evidence to Marris’ analyses of an unequal distribution of uncertainty in Western societies, namely within work environments.

The model better explains emotional coping strategies since cognitive uncertainty’s variance is only explained by 37%, and desire for change’s variance by 12%. PS-US’s subscale with a higher predictive value of cognitive uncertainty is self-defeating beliefs, followed by psychosocial consequences at work and, with the smallest contribution, psychosocial consequences within relationships and communities. Even though the scale can explain this variable, it is clearly lower than its effect on emotional uncertainty. So, we can conclude that PS-US will better explain emotional responses to uncertainty than strategies of planning and control, which may be less influenced by individuals’ perception and experience of psychosocial uncertainty. On the other hand, the fact that self-defeating beliefs better explains cognitive uncertainty may be understood if we consider that both represent an anticipation towards the future, whether through beliefs and expectations, whether through planning and control.

Regarding desire for change, analysing each of the subscales’ contribution, the only variable that significantly contributes to desire for change’s variance is self-defeating beliefs. This result may be understood by considering that desire for change reflects a positive perspective on change and uncertainty, while PS-US’ dimensions reflect psychosocial uncertainty and its consequences, which are experienced more intensely by individuals in vulnerable conditions, and so, are perceived as negative. So, it is understandable that this scale does not contribute as expressively to explain desire for change. The fact that self-defeating beliefs is the variable that better explains desire for change can be understood if we consider the correlation results between these dimensions. Therefore, these variables may work in a similar but opposite fashion: self-defeating beliefs is associated with underprivileged groups, and desire for change, by reflecting an availability towards it, may associate with privileged groups. So, this result supports Marris’ social analysis of uncertainty as unequally distributed, leading different social groups to develop different resources and strategies to deal with uncertainty.

Moreover, the dimensions of PS-US may express individuals’ attitudes towards reality, the world and others, which reflect models of representation of reality developed throughout the living cycle, from life experiences and attachment relationships, and so, from internal working models and self-representation models (Bowlby, 1980). Thus, the way the individual understands, conceptualises, and creates meaning for uncertainty influences the coping strategies developed to deal with it, having an emotional effect. So, past positive experiences will have a crucial role in how one gives meaning to uncertainty, as well as the support context one has, when dealing with it. However, if the situation or environment is too challenging for the support structure available, this experience may be destabilising, affecting the ability to assess the uncertain situation and the personal resources to cope with it.

On the other hand, we must take into account the importance of meaning-making for psychological internal coherence when facing pressure for change, since a new equilibrium is needed for self-continuity and for maintaining internal consistency when assimilating new experiences and meanings. In a social context strained with the consequences of individualisation, globalisation, isolation, distrust, among others, uncertainty may challenge individuals in a new manner. So, these new forms of uncertainty seem to complicate the efforts of understanding oneself and giving meaning to personal experiences, the world and others.

## Discussion

This study contributes to the development of a new measure that may allow the assessment of psychosocial uncertainty. Its creation process, based on Marris’ (1991, 1996) proposal, as well as on contributions from various backgrounds, is presented, along with several steps of its psychometric assessment: factor structure, the scale’s validity and reliability, group invariance between two samples and across gender, SCL levels and group of origin, group differences in demographical variables, associations with the URS and a model that represents the relationship between these two scales. Women and individuals from lower SCL were expected to experience greater psychological consequences of psychosocial uncertainty in all its dimensions. The PS-US was expected to present moderate and weak correlations with the URS, providing evidence for both scales’ divergent validity. Furthermore, it was hypothesised that coping with uncertainty could be explained by psychosocial uncertainty, following Marris’ proposition of an unequal distribution of uncertainty and the existence of forms of uncertainty that are socially created. Through these analyses we also intended to propose the combined use of PS-US and URS to explore the relationship between psychosocial uncertainty and strategies of coping with uncertainty.

Validation results from the development of PS-US show that this scale is reliable and valid, conveying a sense of quality, with acceptable internal consistency values and composite reliability levels (Borsboom et al., 2004). Despite limitations in the third dimension of PS-US, which should be further developed, the scale proves to be a useful instrument in the assessment of psychosocial uncertainty. AVE achieved satisfactory levels for an exploratory study, providing support for divergent reliability. Results reveal criterion concurrent validity, as it proved to be able to differentiate groups regarding experiences of uncertainty. The preliminary CFA allowed to assess the scale’s structure and psychometric qualities, confirming them through the good fit found in the second CFA via a shorter version.

Multi-group measurement invariance analysis proved PS-US’ full configural and metric invariance, and partial scalar invariance if the Δ RMSEA and Δ SRMR results are considered (Cheung & Rensvold, 2002), providing partial psychometric support to the comparability of cross-sectional studies. Similarly, gender invariance achieved full metric invariance and partial scalar invariance. However, gender comparison results must be cautiously judged, and further invariance studies should verify multi-group and gender invariance.

Concerning the process of development of this scale, based on Marris’ theoretical proposal (1996), these items may reflect structures of meaning of uncertainty. Since they articulate and relate public meanings (that result from the process of construction of collective meanings, which are based on the categorisation of the world, reflecting abstract structures of interpretation of reality), with personal meaning (individual and developed through attachment relationships throughout the life cycle) (Marris, 1996). Therefore, they may reflect meta-meanings since they transmit perceptions, beliefs, feelings and concerns (and so personal meanings) that are contextualised within social situations (unemployment), the abstract other (distrust) or the future (self-defeating beliefs), which are set within public meanings. In this sense, meta-meanings, by relating to different kinds of meanings, may promote the organisation of behaviour and situations into consistent patterns to choose how to understand them, make them more predictable or manageable and, so, cope with them. This allows us to grasp the complexity of the psychological construct here studied, since it seeks this articulation between public and personal meanings. So, the process of negotiation between public and personal meanings influences how people will understand a specific event/person and react towards it. However, power affects this process of negotiation, since I may or may not have power to reject (or accept) a specific system of public meaning. Considering that social circumstances are the foundation for the social power one has, these will be crucial in how people cope with uncertainty.

As hypothesised, underprivileged or vulnerable social groups (women, lower SCL) presented higher levels of psychosocial uncertainty, experiencing its consequences in a more dramatic way in their relationships, work and a greater tendency to demonstrate self-defeating beliefs towards uncertainty (Bauman, 2001; Marris, 1996). Moreover, professionals also seem to experience higher levels of psychosocial uncertainty than students, which suggests that the labour market is characterised by uncertainty in many forms (job insecurity, employment uncertainty, precarity…) (de Witte et al., 2016; Mantler et al., 2005; Mohr, 2000; Standing, 2011).

Furthermore, results show that these scales complement each other since the associations found between the scales give evidence that the adoption of emotional maladaptive strategies to cope with uncertainty is associated with psychosocial uncertainty (and may be affected by it). The effects of sociodemographic variables (gender, SCL, group of origin) that were found reinforce the thesis that social contexts create uncertainty that is perceived and dealt with differently by people, according to their social, economic and cultural circumstances. Considering that previous research found these same differences for gender and SCL concerning emotional uncertainty with the URS (Lucas Casanova et al., 2019), these results suggest that the use of emotional coping strategies should be understood as a consequence of living circumstances and not simply as a psychological trait and, therefore, as a consequence of socially created uncertainty and as a self-defeating strategy people are led to use when they are powerless towards uncertainty. So, these scales offer the opportunity to explore new forms of uncertainty in Western contemporary societies and how they constrain people’s lives.

Nevertheless, it is worth mentioning that this is a cross-sectional study, using a convenience sample, limiting conclusions in terms of generalisation due to unbalanced distributions of groups, and causality interpretations. Therefore, further studies may concentrate on additional analyses in terms of multi-groups and gender invariance, and longitudinal invariance and predictive validity, exploring this potential causality longitudinally. Despite these limitations, results show that this scale might become valuable for the analysis of psychosocial uncertainty and its consequences, and it would be interesting to explore the relationship of psychosocial uncertainty and coping with uncertainty with other variables, such as the experience of employment (or unemployment), or attachment relationships.

To the best of our knowledge, up to this point, there were no measures that approached uncertainty in this manner, considering Tomasik and Silbereisen’s Scale (2009) focuses on demands of social change (which entail uncertainty) but was not developed as an uncertainty scale, and that other authors focus on specific aspects of social uncertainty, such as economic uncertainty (Griskevicius et al., 2011, 2013; Mittal et al., 2015; Mittal & Griskevicius, 2014). Moreover, we propose that the combined use of PS-US and URS may foster a more integrated understanding of this phenomenon. So, besides psychological dimensions that lead people to use self-defeating strategies, by reducing their sense of control (Mittal & Griskevicius, 2014), it is important to stress that social conditions of vulnerability (lower SES, schooling levels, etc.) actually constrain individuals’ power to cope with environmental threats such as uncertainty, undermining people’s and communities’ agency. Therefore, their perceptions of control may be realistic in their negativity, even if the beliefs they generate may contribute to a sense of uncontrollability and despair and, consequently, create conditions for failure. Therefore, psychological intervention in these matters should not focus solely on helping individuals increase their perception of control since, if their social circumstances do not change, that perception would be unrealistic. In this sense, we hope the joint use of these scales may be useful for research, so that they may help identify forms of intervention that may in fact ascribe individuals and communities with more possibilities for agency and for controlling their environment.

## Conclusions

This study contributes to quantitative research on the psychological dimension of uncertainty, relating it with social conditions, in order to foster a greater interest in the research of psychosocial dimensions of uncertainty. A solely psychological approach to uncertainty, by reinforcing an intrapsychic understanding of a phenomenon that has historical, social and cultural origins, may, unwillingly, generate discourses as well as social, psychological or political interventions that are merely focused on transforming the individual and fostering its adaptation to the environment. By doing so, we become accomplices in the reproduction of social discourses on the inadequacy of the most vulnerable, which increase the uncertainty they face and further constrain their power to control it (Marris, 1996), falling into the trap of victim blaming (Ryan, 1971/1976), while obliterating the importance of context transformation (Prilleltensky, 1994).

## Supplementary Information

**Additional file 1: Supplementary Material** includes tables and figures identified in the manuscript, as well as correlations and covariances matrices.

## Data Availability

The datasets generated during and/or analysed during the current study are available from the corresponding author on reasonable request.

## Abbreviations

AVE: Average variance extracted
CFA: Confirmatory factor analysis
CFI: Comparative Fit Index
df: degrees of freedom
EFA: Exploratory factor analysis
IA: Intolerance of ambiguity
IU: Intolerance of uncertainty
Ku: Kurtosis
m.v.: missing values
MECVI: Model Expected Cross Validation Index
p: *P* value
PAF: Principal axis factoring
PS-US: Psychosocial Uncertainty Scale
RMSEA: Root mean square error of approximation
SCL: Sociocultural level
Sk: Skewness
SRMR: Standardised root mean square residual
TLI: Tucker-Lewis Index
URS: Uncertainty Response Scale
χ2: Chi-square

## Footnotes

1 Despite the items’ positive formulation, given the fact that the rest of the scale was negatively worded, these items are inverted, as well as item 52.
