# Supplementary material for: Factorial validity and measurement invariance of the Psychosocial Uncertainty Scale
Source: Psicol Reflex Crit. 2021 Jul 30;34:25. doi: 10.1186/s41155-021-00190-z (PMC8324646; doi:10.1186/s41155-021-00190-z)
Supplement: Supplementary file 1 — Additional file 1: Supplementary Material includes tables and figures identified in the manuscript, as well as correlations and covariances matrices. [file 41155_2021_190_MOESM1_ESM.docx]

Appendix A

Descriptive statistics for the PS-US (Portuguese Version)

|  |  | **EFA Sample (N=827)** | | | | | | | | **CFA Sample (N=382)** | | | | | | **MG Sample (CFA2) (N=387)** | | | | | |  |
| --- | --- | --- | --- | --- | --- | --- | --- | --- | --- | --- | --- | --- | --- | --- | --- | --- | --- | --- | --- | --- | --- | --- |
| **Items’**  **Number** | **Items’**  **Label** | **Range** | **Min** | **Max** | **M** | **Md** | **SD** | **Sk** | **Ku** | | **M** | **MD** | **SD** | **Sk** | **Ku** | | **M** | **Md** | **SD** | **Sk** | **Ku** | |
| 1 | ISoc1 | 4 | 1 | 5 | 3.74 | 4.00 | 1.04 | -0.78 | 0.26 | | 3.78 | 4.00 | 1.05 | -0.85 | 0.44 | | 3.78 | 4.00 | 1.04 | -0.79 | 0.31 | |
| 2 | ISoc4 | 4 | 1 | 5 | 2.31 | 2.00 | 0.98 | 0.49 | -0.15 | | 2.33 | 2.00 | 1.01 | 0.69 | 0.30 | | 2.40 | 2.00 | 1.05 | 0.57 | -0.15 | |
| 3 | ISoc8 | 4 | 1 | 5 | 3.53 | 4.00 | 1.23 | -0.47 | -0.75 | | 3.38 | 3.00 | 1.31 | -0.31 | -1.03 | | 3.44 | 4.00 | 1.26 | -0.38 | -0.91 | |
| 4 | ISoc11 | 4 | 1 | 5 | 3.12 | 3.00 | 1.14 | -0.22 | -0.70 | | 3.14 | 3.00 | 1.13 | -0.15 | -0.77 | | 3.09 | 3.00 | 1.12 | -0.12 | -0.78 | |
| 5 | ISoc15 | 4 | 1 | 5 | 2.78 | 3.00 | 1.06 | 0.13 | -0.66 | | 2.74 | 3.00 | 1.10 | 0.09 | -0.77 | | 2.79 | 3.00 | 1.08 | 0.09 | -0.68 | |
| 6 | ISoc17 | 4 | 1 | 5 | 3.27 | 3.00 | 1.19 | -0.28 | -0.83 | | 3.28 | 3.00 | 1.25 | -0.29 | -0.97 | | 3.24 | 3.00 | 1.19 | -0.39 | -0.68 | |
| 7 | ISoc20 | 4 | 1 | 5 | 2.94 | 3.00 | 1.08 | -0.01 | -0.69 | | 2.98 | 3.00 | 1.14 | -0.05 | -0.74 | | 2.89 | 3.00 | 1.09 | -0.03 | -0.80 | |
| 8 | ISoc22 | 4 | 1 | 5 | 2.56 | 2.00 | 0.99 | 0.35 | -0.23 | | 2.62 | 3.00 | 1.03 | 0.31 | -0.44 | | 2.56 | 3.00 | 1.01 | 0.23 | -0.38 | |
| 9 | ISoc26 | 4 | 1 | 5 | 3.84 | 4.00 | 1.14 | -0.72 | -0.36 | | 3.86 | 4.00 | 1.17 | -0.83 | -0.22 | | 3.87 | 4.00 | 1.15 | -0.80 | -0.24 | |
| 10 | ISoc31 | 4 | 1 | 5 | 4.05 | 4.00 | 1.00 | -1.07 | 0.81 | | 3.95 | 4.00 | 1.04 | -0.94 | 0.46 | | 4.07 | 4.00 | 0.96 | -1.02 | 0.63 | |
| 11 | ISoc34 | 4 | 1 | 5 | 2.69 | 3.00 | 1.07 | 0.17 | -0.50 | | 2.70 | 3.00 | 1.02 | 0.19 | -0.43 | | 2.71 | 3.00 | 1.06 | 0.25 | -0.44 | |
| 12 | ISoc38 | 4 | 1 | 5 | 3.10 | 3.00 | 1.07 | -0.07 | -0.45 | | 3.12 | 3.00 | 1.04 | -0.08 | -0.46 | | 3.11 | 3.00 | 1.04 | -0.20 | -0.35 | |
| 13 | ISoc41 | 4 | 1 | 5 | 2.75 | 3.00 | 1.12 | 0.09 | -0.71 | | 2.76 | 3.00 | 1.09 | -0.02 | -0.82 | | 2.75 | 3.00 | 1.08 | 0.02 | -0.65 | |
| 14 | ISoc43 | 4 | 1 | 5 | 3.16 | 3.00 | 1.03 | -0.09 | -0.48 | | 3.19 | 3.00 | 1.04 | -0.17 | -0.46 | | 3.26 | 3.00 | 1.03 | -0.18 | -0.54 | |
| 15 | ISoc46 | 4 | 1 | 5 | 2.41 | 2.00 | 0.89 | 0.50 | 0.26 | | 2.31 | 2.00 | 0.87 | 0.81 | 0.72 | | 2.47 | 2.00 | 0.94 | 0.51 | 0.23 | |
| 16 | ISoc47 | 4 | 1 | 5 | 2.05 | 2.00 | 0.95 | 0.62 | -0.28 | | 2.08 | 2.00 | 0.96 | 0.72 | 0.09 | | 2.07 | 2.00 | 0.94 | 0.68 | 0.04 | |
| 17 | ISoc49 | 4 | 1 | 5 | 2.88 | 3.00 | 0.98 | 0.30 | -0.17 | | 2.84 | 3.00 | 1.01 | 0.23 | -0.29 | | 2.95 | 3.00 | 0.99 | 0.24 | -0.38 | |
| 18 | ISoc52 | 4 | 1 | 5 | 3.03 | 3.00 | 0.87 | 0.28 | 0.07 | | 3.00 | 3.00 | 0.94 | 0.31 | -0.14 | | 3.09 | 3.00 | 0.90 | 0.21 | -0.19 | |
| 19 | ISoc56 | 4 | 1 | 5 | 1.71 | 2.00 | 0.82 | 1.13 | 1.32 | | 1.74 | 2.00 | 0.84 | 1.19 | 1.61 | | 1.74 | 2.00 | 0.83 | 1.09 | 1.15 | |
| 20 | ISoc59 | 4 | 1 | 5 | 3.27 | 3.00 | 1.10 | -0.19 | -0.52 | | 3.34 | 3.00 | 1.08 | -0.36 | -0.38 | | 3.30 | 3.00 | 1.16 | -0.32 | -0.60 | |
| 21 | ISoc65 | 4 | 1 | 5 | 2.88 | 3.00 | 1.10 | 0.08 | -0.62 | | 2.76 | 3.00 | 1.17 | 0.19 | -0.76 | | 2.83 | 3.00 | 1.11 | 0.09 | -0.65 | |
| 22 | ISoc67 | 4 | 1 | 5 | 3.37 | 3.00 | 0.98 | -0.29 | -0.14 | | 3.54 | 4.00 | 0.99 | -0.35 | -0.23 | | 3.41 | 4.00 | 0.99 | -0.43 | -0.13 | |

*Note.* Min= minimum; Max=maximum; M=mean; Md=Median; SD=standard deviation; Sk=Skewness; Ku=Kurtosis

Appendix B

Table B1.

EFA - PAF with Oblique rotation (.4) – URS Distribution with item loadings

| Items' Number | Items' Label | Work | Relationships | Beliefs |
| --- | --- | --- | --- | --- |
| 3 | ISoc8 | .80 |  |  |
| 7 | ISoc20 | .54 |  |  |
| 8 | ISoc22 | .39 |  |  |
| 9 | ISoc26 | .41 |  |  |
| 10 | ISoc31 | .75 |  |  |
| 21 | ISoc65 | .44 |  |  |
| 4 | ISoc11 |  | .96 |  |
| 6 | ISoc17 |  | .36 |  |
| 12 | ISoc38 |  | .66 |  |
| 14 | ISoc43 |  | .73 |  |
| 18 | ISoc52 |  | -.32 |  |
| 15 | ISoc46 |  |  | .76 |
| 17 | ISoc49 |  |  | .68 |

Table B2.

*PS-US – eigenvalues, variance explained*

| **Factor** | **Eigenvalues** | **Variance Explained** |
| --- | --- | --- |
| 1 (Work) | 4.38 | 33.69 |
| 2 (Relationships) | 1.52 | 11.70 |
| 3 (Beliefs) | 1.32 | 10.12 |

Table B3

*PS-US – mean, standard deviation and correlations between factors (with EFA sample)*

| **Factor** | **Mean** | **Std. Deviation** | **1** (Work) | **2** (Relationships) |
| --- | --- | --- | --- | --- |
| 1 (Work) | 19.8 | 4.65 |  |  |
| 2 (Relationships) | 15.7 | 3.63 | .50^**^ |  |
| 3 (Beliefs) | 5.29 | 1.62 | .33^**^ | .21^**^ |

*Note.* ^**^ p<.001

Appendix C


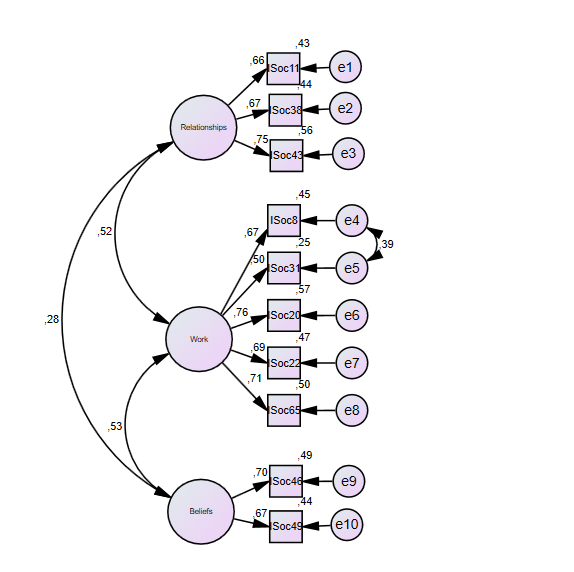


Figure C1. CFA1 - Final PS-US Distribution (Sample 2); standardized coefficients


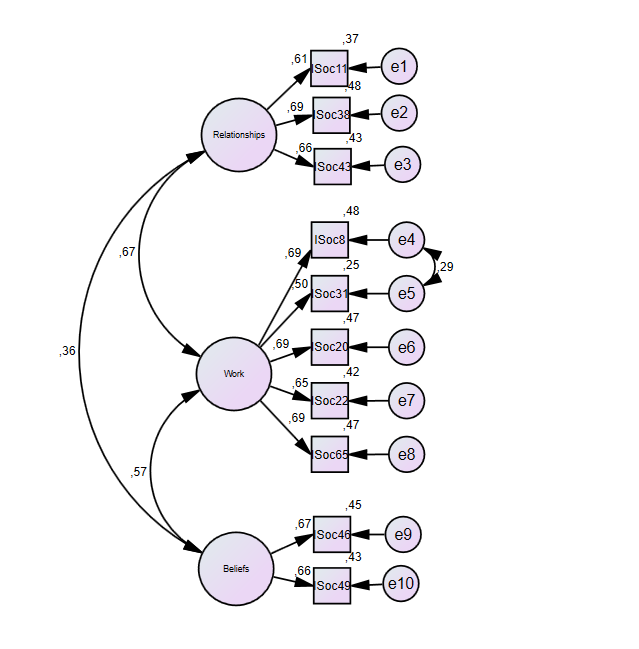


Figure C2. CFA2 - Final PS-US Distribution (Sample 3); standardized coefficients

| Table C1  *Distribution and standardized regression weights for the final version of PS-US – CFA 1 (Sample 2)* | | | |
| --- | --- | --- | --- |
|  | **Item** | **Standardized Regression Weights** |  |
|  | 3. *When I hear about unemployment rates increasing, I worry about my future* / Quando oiço falar do aumento das taxas de desemprego, fico preocupado(a) com o meu futuro. | .67 |  |
| Psychosocial consequences at work | 7. *Because of the characteristics of the labour market, I feel increased difficulties in making decisions* / Considerando as características do mercado de trabalho sinto cada vez mais dificuldades a tomar decisões. | .76 |  |
|  | 8. *I feel lost before the world’s constant changes* / Sinto-me perdido(a) perante as constantes mudanças no mundo atual. | .69 |  |
|  | 10. I *worry with nowadays’ reduction of job positions* / Preocupo-me com o atual encerramento de postos de trabalho. | .50 |  |
|  | 21. *I feel I cannot plan my career because of the economic crisis*. / Sinto que não posso planear a minha carreira por causa da crise económica. | .71 |  |
|  | 4. *The way life is organised in cities makes me think I cannot rely on others when I’m in trouble* / A forma como a vida está organizada nas cidades, faz-me pensar que não posso contar com os outros quando estou em dificuldades | .66 |  |
| Psychosocial consequences relationships/communities | 12. *I increasingly feel the world that surrounds me as hostile* / Cada vez mais sinto o mundo que me rodeia como hostil. | .67 |  |
|  | 14. *The competition that exists in nowadays societies makes me feel I cannot trust others* / A competição que existe entre as pessoas na sociedade atual faz-me sentir que não posso confiar nos outros. | .75 |  |
| Self-defeating Beliefs | 15. *Despite the unpredictability of contemporary life, I feel I can plan my future /* Apesar da imprevisibilidade do mundo atual, sinto que sou capaz de planear o meu futuro. | .70 |  |
|  | 17. *I believe I can control my future* / Acredito que posso controlar o meu futuro. | .67 |  |
|  |  |  |  |

*Note.* Free translation from the Portuguese items
